# Chronic semaglutide alters ingestive behavior without impairing taste function in mice

**DOI:** 10.1016/j.molmet.2026.102410

**Published:** 2026-06-27

**Authors:** Alisha A. Acosta, Hillary Ellis, Fang-Yu Hsu, Karen K. Yee, Guillaume de Lartigue, Amber L. Alhadeff

**Affiliations:** 1 Monell Chemical Senses Center, Philadelphia PA 19104, USA; 2 Department of Neuroscience, University of Pennsylvania, Philadelphia PA 19104, USA

**Keywords:** GLP-1R, semaglutide, taste, obesity, sucrose

## Abstract

Glucagon-like peptide-1 receptor (GLP-1R) agonists such as semaglutide are highly effective treatments for obesity, yet the mechanisms by which they reduce food intake remain incompletely understood. Because taste plays a critical role in guiding food intake, several clinical studies have investigated whether GLP-1R agonists alter taste function, but these reports have yielded conflicting results. Here, we systematically tested the effects of chronic semaglutide treatment on taste responsivity in diet-induced obese mice. Mice were evaluated using brief-access gustometer tests to assess responses to sweet, bitter, sour, salty, and fatty tastants. Chronic semaglutide treatment produced robust weight loss but did not alter lick rates for any tastant, indicating intact taste-driven orosensory evaluation across modalities. Psychophysical analysis using a broad range of sucrose concentrations revealed similar concentration-response functions and comparable EC50 values between vehicle- and semaglutide-treated mice, demonstrating unchanged sweet taste sensitivity. However, semaglutide modestly increased total licking and trial initiation for sucrose, suggesting enhanced behavioral engagement rather than altered taste perception. Consistent with the behavioral findings on taste, semaglutide did not affect the abundance of taste receptor cell subtypes in the circumvallate papillae or the expression of genes involved in taste receptor signaling and neurotransmission. Together, these results indicate that chronic semaglutide does not detectably impair peripheral taste function in mice under our experimental conditions. Instead, GLP-1R agonists likely influence ingestive behavior through mechanisms independent of taste signaling, potentially involving alterations in motivational processes.

## Highlights

• GLP-1R agonists robustly suppress body weight without impacting taste function in mice.
• Semaglutide does not alter lick rates for sweet, bitter, sour, salty, or fatty tastants.
• Sweet taste sensitivity is preserved with unchanged concentration-response curves and EC50 values.
• Semaglutide paradoxically increases sucrose-directed behavioral engagement and total intake.
• Semaglutide does not impact taste receptor cell populations or gene expression.

## 1 Introduction

Glucagon-like peptide-1 receptor (GLP-1R) agonists – especially long-acting drugs such as semaglutide (Ozempic/Wegovy) – have transformed obesity treatment. It is widely appreciated that the suppression of body weight occurs largely due to reduced food intake, which results from reduced appetite and increased satiety [1]. While it is known that GLP-1R agonists engage brain pathways involving food motivation, reward, and sickness/malaise [2,3], the complex mechanisms by which these drugs impact eating behavior are still not fully understood.

One key aspect of eating behavior is taste signaling, which provides important information about the quality of food [4]. Taste contributes to food evaluation and ingestive behavior, although its role in the long-term regulation of food intake remains unclear. This raises the question of whether GLP-1R agonists may influence feeding behavior and body weight in part through changes in taste signaling.

Given that GLP-1 and GLP-1R are expressed in taste cells and taste-bud-innervating nerve fibers, respectively [5], previous studies have examined whether GLP-1R signaling can impact taste. GLP-1R knockout mice have reduced sensitivity to sugar, sweeteners, and umami taste, but minimal changes in response to bitter, sour, or salty stimuli [[5], [6], [7]]. Additionally, the first-generation, short-acting GLP-1R agonist exendin-4 reduced lick trials and responses to sugar and fat solutions in rats [8]. By contrast, a recent study suggests that chronic semaglutide treatment robustly increases, rather than decreases, intake of low concentrations of sucrose in rats [9]. It is important to note, however, that changes in number of licks or overall intake may reflect changes in motivation rather than taste signaling.

Furthermore, data from humans on GLP-1R signaling and taste have generated mixed results. One study demonstrated that individuals taking GLP-1R agonists have significantly impaired taste function, and this effect was robust and consistent across all five tested taste modalities [10]. However, two studies showed the opposite: a randomized clinical trial reported that semaglutide improved overall taste recognition, with the largest effect sizes observed in response to sweet and salty tastes [11], and a longitudinal study showed that GLP-1R agonist treatment increased sweet and bitter sensitivity [12]. An additional study analyzed real-world, self-report data which suggested that most individuals do not experience perceived changes in taste [13]. Although methodological differences may explain some of the conflicting reports, there remains no consensus on whether and how GLP-1R agonists influence taste.

Among these discrepant reports, there are no animal studies emulating clinical semaglutide dosing and measuring taste responsivity in brief-access tests. Therefore, we addressed this gap by performing a systematic, controlled study in mice. We put mice on a diet-induced obesity protocol for three months to induce weight gain and subsequently gave chronic injections of semaglutide, with an initial dose escalation followed by maintenance dosing for three weeks. We then examined responsivity to sweet, bitter, sour, salty, and fatty tastants using a brief-access licking protocol and examined gene and protein expression of taste-related molecules. While semaglutide impacted total intake of sugar solutions, overall, our data revealed no detectable changes to taste signaling by semaglutide under our experimental conditions.

## 2 Materials and methods

### 2.1 Animals

Adult male C57BL6/J diet-induced obese (DIO) mice were purchased from Jackson Labs (380050). Mice were group housed (maximum of five mice per cage) on a 12 h:12 h light:dark cycle at 22 °C and 55% humidity on average, with ad libitum access to 60% kcal/fat HFD (Research diets, D12492) and water, unless otherwise noted. Mice were at least 12 weeks old at the start of experimentation. All procedures were approved by the Monell Chemical Senses Center Institutional Animal Care and Use Committee.

### 2.2 Drugs and reagents

Semaglutide (acetate) (Cayman, 29969) was purchased from Cayman and diluted in 0.24% dimethyl sulfoxide (DMSO, Bachem, D8418) in saline. Sucrose (Sigma, S9378), Microlipid (McKesson, 315673) quinine hydrochloride (QHCl, Sigma, Q1125), sodium chloride (NaCl, Spectrum, S0155), and hydrochloric acid (HCl, Sigma, H9892) were diluted in distilled water to the concentrations described under “Brief Access Gustometer Testing” below.

### 2.3 Drug administration

Across all experiments, mice were randomized into control and experimental groups, controlling for initial body weight. Because clinical populations begin treatment with a dose escalation, the experimental groups received daily subcutaneous semaglutide injections (40, 80, 120, 120, and 120 μg/kg) for 5 consecutive days, followed by a maintenance dose (120 μg/kg) once every three days for the duration of the experiment. Control mice received a vehicle (0.24% DMSO in saline) injection whenever experimental mice received a semaglutide injection. Behavioral, histological, and qPCR experiments began after at least 3 weeks of maintenance dosing.

### 2.4 Brief-access gustometer testing

Taste-guided licking was assessed using Davis Rig MS160 mouse gustometers (DiLog Instruments, now Med Associates Inc.). Each apparatus consisted of a test chamber (14.5 × 15 × 15 cm) equipped with a motorized shutter controlling stimulus access and a computer-controlled rack positioning stimulus bottles at the drinking aperture. Licks were detected via an electrical contact circuit integrated into the spout. Mice were trained and tested using a brief-access licking paradigm similar to that in Taruno et al. (2013) [14] and as described below. Body weight was recorded daily throughout the protocol.

#### 2.4.1 Training

Mice were water restricted for 22.5 h/day. During the initial training session, the shutter remained open for 30 min to allow continuous access to water, followed by 1 h of water in the home cage. In subsequent sessions, water was delivered in 5-s trials. If no lick occurred, the shutter closed after 5 s; if licking was initiated, it remained open for 5 s from the first lick. Each trial was followed by a 7.5-s inter-presentation interval (IPI). Training sessions lasted 20 min and continued for 3–4 days.

#### 2.4.2 Testing

In all test sessions, stimuli were delivered in 5-s access trials followed by a 7.5-s IPI. Sessions lasted 20 min unless otherwise noted. The testing was run across multiple independent groups of mice. Each group included semaglutide-treated and vehicle-treated mice injected every 3 days. Testing sessions were coordinated so that the effects of injection timing could be assessed as an additional variable.

For sucrose or fat responsivity testing, mice were ad libitum fed except for in the 24 h prior to testing, where they received 1 g of food and 2 mL of water. During testing, mice received presentations of varying concentrations of sucrose (0, 32, 100, 320, 1000 mM) or fat (0, 2, 4, 8, or 16% Microlipid). Sessions began with a single presentation of the highest concentration to initiate responding. Thereafter, repeated series of five concentrations (including water as 0) were presented in quasi-random order such that each concentration appeared once per series. Testing occurred 24 h, 48 h, or 72 h after semaglutide or vehicle injection, and each mouse received three test sessions at each time point from which we averaged response data. To avoid excess testing in each mouse, and to keep the duration of chronic semaglutide injections relatively consistent across data, each post-injection timepoint was run in separate cohorts of mice. In an additional cohort of mice, a larger range of sucrose (0, 0.32, 1, 3.2, 10, 32, 100, 320, 640, 1000 mM) was provided to better measure sweet taste sensitivity. All parameters for this group were identical to those described above except that sessions lasted 30 min to permit an adequate number of presentations of each concentration from the expanded range, and each mouse completed four sessions.

For tastants that are aversive at high concentrations [QHCl (0, 0.032, 0.1, 0.32, 1 mM), NaCl (0, 150, 300, 600, 1000 mM), and HCl (0, 1, 3.2, 10, 32 mM)], mice received 1 h of water in the home cage following each session and were then water restricted (22.5 h) before the next session. Five concentrations (including water as 0) were presented in repeated randomized series. To minimize carryover effects and maintain responding, 1-s water rinse trials were interposed between tastant presentations. Thus, each 5-s tastant trial was followed by a 7.5-s intertrial interval, a 1-s water trial, and an additional 7.5-s interval before the next trial. Mice were tested at 24 h, 48 h, and 72 h post-injection and responses were averaged across timepoints.

#### 2.4.3 Gustometer data analysis

The lick rates and total licks for each concentration of each tastant for individual mice were averaged across at least three sessions, based on recommendations of previous brief-access taste test studies [15,16]. Similarly, the total number of licks and total trials completed were summed for each mouse and averaged across all sessions. For the sucrose taste sensitivity test, we normalized average lick rate to the maximum lick rate for each individual mouse to isolate taste evaluation from motivation. We then fit data with a non-linear regression, using a four-parameter dose–response curve [17]. The upper asymptote was capped at 1.0 (the mouse's maximum lick rate). To calculate EC50 and area under the curve for each mouse, we fit each mouse's data with non-linear regression using the same criteria. One mouse was removed from this analysis due to an unstable curve fit.

### 2.5 Immunohistochemistry

#### 2.5.1 Tissue and processing

Mice were treated with chronic vehicle or semaglutide injections as described above. After 4% paraformaldehyde fixation, the circumvallate papillae (CvP) were dissected from tongue tissue, post-fixed for 1–2 h at 4 °C, and cryoprotected in 10%, 20%, and 30% sucrose serially overnight at 4 °C. Tissues were frozen in OCT (Electron Microscopy Sciences). Coronal cryosections (8 μm) were cut through the entire CvP, mounted onto SuperFrost Plus slides (Electron Microscopy Sciences) and stored at −80 °C until use. For reference data, CvP tissue from additional cohorts of age-matched chow and high-fat diet-fed mice (3 months on diet, without drug injections) was prepared exactly as described above.

#### 2.5.2 Immunohistochemical staining

CvP sections were rehydrated with ddH_2_O and incubated in 80 °C preheated Dako target retrieval solution, pH 9 (Agilent) for 20 min. 5% normal donkey serum (Sigma, D9663) in phosphate buffer, pH 7.4 with 0.1% Triton X-100, was used to block nonspecific sites. Sections were incubated overnight at 4 °C with rabbit anti-taste receptor type I member 3 (T1R3, 1:1000, Ohmoto et al., 2008), goat anti-carbonic anhydrase IV (CAR4, 1:1000, R&D systems, AF2414, RRID: AB_2070332) and mouse anti-inositol 1,4,5-triphosphate receptor, type 3 (ITPR3, 1:1000, BD Biosciences, 610,312, RRID: AB_397704) primary antibodies. After washing in PBS, sections were incubated for 1 h at room temperature with Alexa Fluor 488 donkey anti-rabbit (1:500, Thermo Fisher Scientific, A-21206), Alexa Fluor 555 donkey anti-goat (1:500, Thermo Fisher Scientific, A-21432) and Alexa Fluor 647 donkey anti-mouse (1:500, Thermo Fisher Scientific, A-31517) secondary antibodies. Cell nuclei were labeled with 4′,6-Diamidino-2-phenylindole dihydrochloride (DAPI, 1:1000, Sigma, D9542).

#### 2.5.3 Imaging and analysis

Immunofluorescence images were captured at 20× magnification on a Leica Stellaris 5 confocal microscope using 448-nm, 555-nm, and 647-nm laser settings, as well as appropriate excitation spectra. Series z-stacks were captured at a step size of 0.69 μm through the entire section. Images were scanned using a 1024 × 1024 pixel format with 2-line averaging.

Approximately 30–40 sections are generated from sectioning an entire CvP. Sections were serially mounted so that each slide would have an anterior, middle and posterior section of the same CvP ∼112 μm apart. Three CvP sections yield a total of 45–50 taste buds. We quantified the immunolabeled taste cells in vehicle- and semaglutide-treated mice. Specifically, in each CvP section, the number of T1R3+, CAR4+, and ITPR3+/T1R3-labeled cells with visible DAPI + labeled nuclei were manually counted in LASX software (Leica). The total cell numbers were determined for each mouse.

### 2.6 Quantitative real-time PCR

Mice were treated with chronic vehicle or semaglutide injections as described above. After euthanasia, total RNA was extracted from CvP using TRIzol (Invitrogen, #15596026) with the PureLink™ RNA Mini Kit (Invitrogen, #12183018 A). 2 μg of total RNA was subjected to reverse transcription with High-Capacity cDNA Reverse Transcription Kit (Applied Biosystems, #43-749-66). Quantitative PCR was performed using KAPA SYBR® FAST (Roche, #KK4605). Relative mRNA expression was determined using the comparative Ct (2^−ΔΔCt^) method. ΔCt values were calculated by normalizing the target gene to *Gapdh* expression. These values were then normalized to the mean of the vehicle control group to determine relative fold change. The primers used are provided in Table 1. For reference data, CvP tissue from additional cohorts of age-matched chow and high-fat diet-fed mice (3 months on diet, without drug injections) was prepared exactly as described above.

**Table 1:** Primers used in qPCR experiment.

Gene	Forward (5′ → 3′)	Reverse (5′ → 3′)
Pkd2l1	TGCCATCGACAATGCCAACAG	CGCAGCCTTAGTAGGGTCTTG
Car4	CAATGGGTCAATGTGCTCTG	GGGGACTGCTGATTCTCCTT
Tas2r105	TGACTGGCTTCCTTCTCATCG	TCAGGTGATTCACAGTCATCC
Tas2r118	GCTGTCCTCTACTGTGTCAAG	AGAAGCTATCAGAGCACCCAG
Pou2f3	TCTCAGCGAAGTGTTTGGCAG	TTGCAGAACCAGACTCTCACC
Tas1r2	TCGTCTATCCATGGCAGCTAC	TAGGAGGCGATGCTTTGGAAG
Tas1r3	ACCTTCAACGGCACCCTTCAG	CAGGTGAAGTCATCTGGATGC
Calhm1	TTGCGCTGCATCTCTCAGGCA	TAGTGGGACCAGTACTTGCTC
Calhm3	GCAAGTGCAGCTCTTCCTAGC	TCCAGCCGATGGCCTGTGACA
Trpm5	GGAAAACGGCACACAGAGTGG	CCACAGTTCTGAGAGCTTGAG
Gnat3	GACTTAGACAGACTCACAGCC	CTCTGATCTCTGGCCACCTAC
Plcb2	CCTGGAGGTGACAGCTTATGA	GAGCTCCGTGAAGGAAGAGAC
Gapdh	GCATGGCCTTCCGTGTTCCTA	GATGCCTGCTTCACCACCTTCT

### 2.7 Statistical analyses

All data were expressed as mean ± S.E.M. Unpaired t-tests, two-way repeated measures ANOVA (with post hoc Šídák's multiple comparisons), and Pearson correlation were performed where appropriate using GraphPad Prism. For the taste sensitivity test, data were fit with a non-linear regression as described above under “Gustometer data analysis.” Sample sizes, statistical tests, and p-values for each experiment are listed in Supplementary Table 1. Ns, p > 0.05; ∗p < 0.05; ∗∗p < 0.005; ∗∗∗p < 0.001.

## 3 Results

### 3.1 Chronic semaglutide does not impair behavioral sweet taste responsivity but increases overall sugar intake

**Figure 1:**
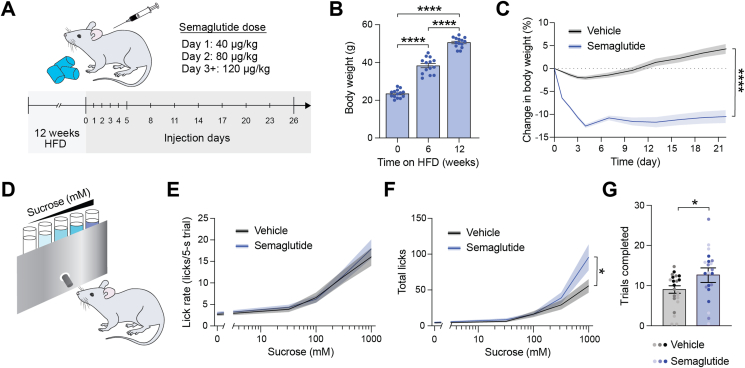
**Chronic semaglutide does not impair sweet responsivity but modestly increases overall sugar intake.** (A) Schematic and timeline for diet-induced obesity and chronic semaglutide injections. Experimentation began after at least 3 weeks of maintenance dosing (after Day 26), and injections continued every 3 days throughout the duration of experiments. (B) Body weight of mice on high-fat diet (HFD) across 12 weeks (n = 14, one-way repeated measures ANOVA, p < 0.0001). (C) Change in body weight in vehicle- or semaglutide-treated mice across 3 weeks of drug treatment (n = 23–24/group, two-way repeated measures ANOVA, p < 0.0001). (D) Schematic for brief-access taste tests where mice received brief exposures to water plus 4 concentrations of sucrose in quasi-random order. (E) Lick rate across sucrose concentrations in vehicle- and semaglutide-treated mice (n = 21–22/group, two-way repeated measures ANOVA, p = ns). (F) Total licks across sucrose concentrations in vehicle- and semaglutide-treated mice (n = 21–22/group, two-way repeated measures ANOVA, p < 0.05). (G) Total trials completed in vehicle- and semaglutide-treated mice (n = 21–22/group, unpaired t-test, p < 0.05). Light dots, medium dots, and dark dots represent mice tested 24 h, 48 h, or 72 h post-injection, respectively. Data are presented as mean ± S.E.M. ns p > 0.05, ∗p < 0.05, ∗∗p < 0.01, ∗∗∗p < 0.001, ∗∗∗∗p < 0.0001. See Supplementary Table 1 for full statistical details.

To determine how chronic semaglutide treatment impacts taste, we first put mice on a high-fat diet for three months for diet-induced obesity (Figure 1A,B). We then began semaglutide treatment: we gave daily injections in escalating doses for 5 days, followed by maintenance dosing for 3 weeks (Figure 1A). Semaglutide treatment caused a robust and sustained reduction in body weight (Figure 1C). While still on semaglutide, we gave mice brief access to water or four concentrations of sucrose in a counterbalanced order and tested their responsivity to these solutions (Figure 1D). Because mice received semaglutide injections every three days, we measured taste responsivity at 24, 48, and 72 h post-injection and averaged these responses for an overall representation of how semaglutide influences taste responsivity. In rodents, lick rate is used as a measure of orosensory/taste evaluation because it reflects rapid taste-driven responding largely independent of motivational and post-ingestive influences [16]. In contrast, total licks and total trials completed across a session incorporate motivational state and metabolic feedback. Compared to vehicle-treated mice, semaglutide-treated mice did not differ in lick rate for sucrose solutions (Figure 1E), indicating that the drug does not cause detectable changes in sweet taste or palatability under our experimental conditions. However, total licks (Figure 1F) and total number of trials completed (Figure 1G) were modestly increased in semaglutide-compared to vehicle-treated mice.

Because semaglutide has a long half-life [18] and can have time-dependent effects on ingestive behaviors [19,20], we next examined licking behavior across different timepoints post-injection. When we examined taste responsivity in sessions 24 h post-semaglutide injection, we observed a slightly decreased lick rate at higher concentrations (Fig. S1A), likely reflecting the nausea/malaise that occurs acutely after semaglutide treatment [21,22]. The number of licks (Fig. S1B and C) and total trials completed (Fig. S1D) were unchanged across groups. In contrast, at both 48 h and 72 h after injection, we observed the opposite trend: while semaglutide treatment did not impact lick rate (indicating no change in sweet taste, Fig. S1E and I), it modestly elevated the total licks and trials completed in each session, effects that were trending at 48 h post-injection (Fig. S1F–H) and reached statistical significance at 72 h post-injection (Fig. S1J-L).

### 3.2 Chronic semaglutide does not impact behavioral sweet taste sensitivity

**Figure 2:**
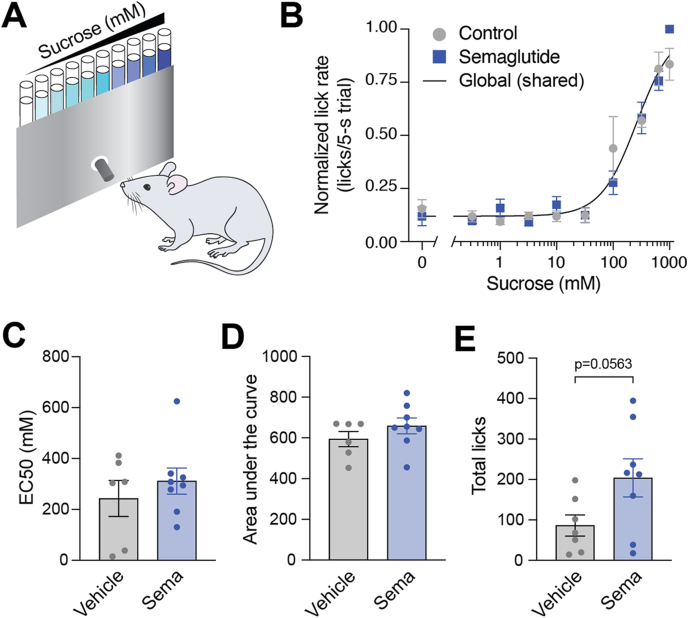
**Chronic semaglutide does not impact sweet taste sensitivity.** (A) Schematic for brief-access taste tests where mice received brief exposures to water plus 9 concentrations of sucrose in quasi-random order. (B) Concentration-response curve in mice treated with vehicle or semaglutide injections. A non-linear regression fit revealed that one curve fits both data sets (n = 7–8/group, p = ns). (C) EC50 of vehicle- and semaglutide-treated mice (n = 6–8/group, unpaired t-test, p = ns). (D) Area under the curve of vehicle- and semaglutide-treated mice (n = 6–8/group, unpaired t-test, p = ns). (E) Total licks in vehicle- and semaglutide-treated mice (n = 7–8/group, unpaired t-test, p = 0.0563). Data are presented as mean ± S.E.M. ns p > 0.05, ∗p < 0.05, ∗∗p < 0.01, ∗∗∗p < 0.001, ∗∗∗∗p < 0.0001. See Supplementary Table 1 for full statistical details.

To directly test whether chronic semaglutide treatment influences sweet taste sensitivity, we subjected a separate group of diet-induced obese, semaglutide-treated mice to a greater number of sucrose concentrations (9 plus water) spanning a logarithmic series (Figure 2A). This approach enabled us to generate concentration-response functions for sucrose and to calculate psychophysical parameters that reflect taste sensitivity. Because semaglutide-treated mice increased overall licking for sucrose in our initial trials, we normalized each mouse's responses to its maximum lick rate. Normalization isolates sucrose sensitivity and hedonics from potential changes in motivation [16].

Vehicle- and semaglutide-treated mice generated nearly identical concentration-response functions across the full range of sucrose concentrations tested (Figure 2B). Quantification of key psychophysical parameters further supported this observation. The half-maximal effective concentration (EC50), which reflects the sucrose concentration required to elicit half-maximal responding and is used as an index of taste sensitivity [17,23], also did not differ between treatment groups (Figure 2C). Similarly, the area under the concentration-response curve, which provides an integrated measure of lick rate across all concentrations tested, was also similar in vehicle- and semaglutide-treated mice (Figure 2D). Consistent with our previous observations, semaglutide caused a slight increase in total licks that was nearly statistically significant (Figure 2E).

We observed considerable variability in sweet taste sensitivity parameters, especially in semaglutide-treated mice. To determine whether this variability stems from individual differences in weight loss on semaglutide, we next correlated each parameter with semaglutide-induced body weight change. Neither EC50, AUC, nor total licks significantly correlated with semaglutide-induced body weight change (Fig. S2A–C). Overall, these findings indicate that chronic semaglutide treatment does not cause detectable changes in behavioral sweet taste sensitivity, and that the variability in sugar responsivity does not significantly relate to body weight change following semaglutide treatment.

### 3.3 Chronic semaglutide does not impair behavioral bitter, sour, salty, or fatty taste responsivity

To determine whether chronic semaglutide treatment affects other taste modalities, we next examined lick responsivity to quinine hydrochloride (QHCl) as a bitter stimulus, hydrochloric acid (HCl) as a sour/acidic stimulus, and sodium chloride (NaCl) as a salty stimulus. These modalities are detected through molecular mechanisms that are largely distinct from those mediating sweet taste and play important roles in guiding ingestive behaviors [4,24]. Because these tastants become strongly aversive at higher concentrations and typically suppress spontaneous licking, mice were tested under water restriction to elevate baseline licking behavior during the brief-access taste tests.

**Figure 3:**
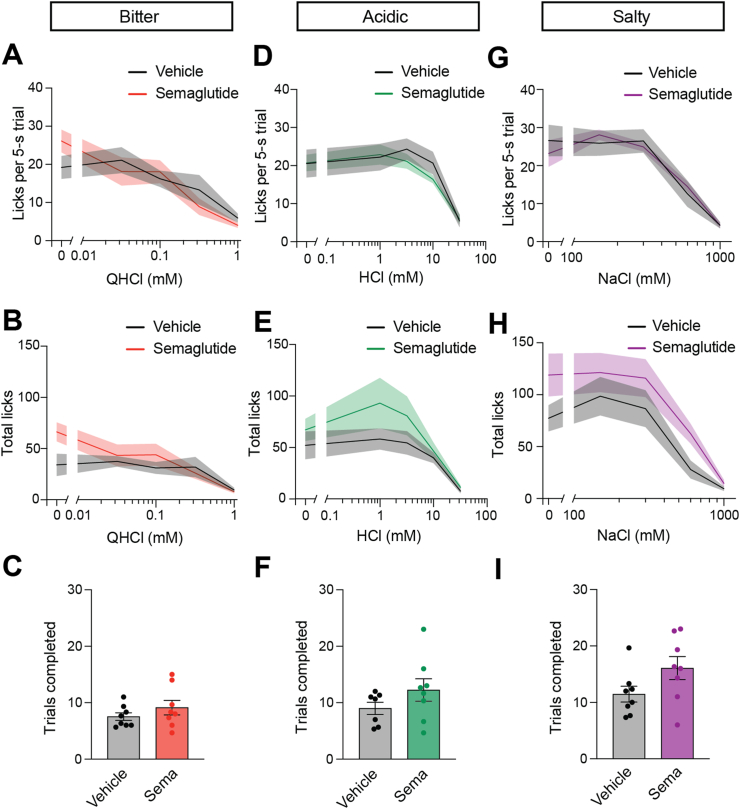
**Chronic semaglutide does not impact responsivity to bitter, acidic, or salty tastants.** (A, D, G) Lick rate across bitter (A, n = 8/group), acidic (D, n = 7–8/group), and salty (G, n = 8/group) concentrations in vehicle- and semaglutide-treated mice (two-way repeated measures ANOVAs, all ps = ns). (B, E, H) Total licks across bitter (B, n = 8/group), acidic (E, n = 7–8/group), and salty (H, n = 8/group) concentrations in vehicle- and semaglutide-treated mice (two-way repeated measures ANOVAs, all ps = ns). (C, F, I) Total trials completed in response to bitter (C, n = 8/group), acidic (F, n = 7–8/group), and salty (I, n = 8/group) tastants in vehicle- and semaglutide-treated mice (unpaired t-tests, all ps = ns). Data are presented as mean ± S.E.M. ns p > 0.05, ∗p < 0.05, ∗∗p < 0.01, ∗∗∗p < 0.001, ∗∗∗∗p < 0.0001. See Supplementary Table 1 for full statistical details.

Across concentrations of bitter, acidic, and salty solutions, chronic semaglutide treatment did not significantly alter taste responsivity. Specifically, semaglutide-treated mice displayed lick rates comparable to those of vehicle-treated controls (Figure 3A, D, G). Similarly, semaglutide did not affect the total number of licks during each session (Figure 3B, E, H) or the number of trials completed (Figure 3C, F, I), indicating that overall task engagement and stimulus-driven licking behavior were unchanged.

Although fat is not traditionally considered a classic taste, accumulating evidence suggests that it shares many primary taste features [25,26]. We therefore asked whether chronic semaglutide influences taste responsivity to fatty solutions. Applying the same behavioral paradigm as we used to test sweet taste responsivity, we showed that semaglutide treated mice did not differ from control mice in lick rate (Fig. S3A and S3D), total number of licks (Fig. S3B and S3E), or number of trials completed (Fig. S3C and S3F) at either the 24 h or 48 h time point tested. Together, these findings suggest that chronic semaglutide does not detectably impair behavioral taste responsivity to bitter, sour/acidic, salty, or fatty stimuli.

### 3.4 Semaglutide does not alter the number of taste cells expressing taste receptors or gene expression of taste receptors and related molecules

Our results suggest that chronic semaglutide treatment does not affect taste responsivity, and so to confirm these behavioral findings, we examined taste tissue for gene and protein expression. Using immunofluorescence labeling, we assessed the number of taste cells expressing markers for sweet/umami-, sour-, and bitter-responsive cells within the CvP, a taste field enriched for cells involved in detection. Specifically, we quantified T1R3^+^ cells which mediate sweet and umami sensing [27], CAR4^+^ cells which mark Type III taste cells associated with sour taste transduction [28], and ITPR3^+^/T1R3^-^ cells as bitter sensing Type II cells [29,30]. We did not label salt- or fat-sensing cells because of the lack of specific markers available.

First, to serve as reference data, we examined the number of taste receptor cells representing distinct taste modalities in mice maintained on chow versus those maintained on high-fat diet. Our data indicated that high-fat diet-fed mice have a reduction in T1R3^+^ cells (Fig. S4A and S4B), which is consistent with gene expression and western blot results in previous reports [31,32]. There were no differences in the number of CAR4^+^ or ITPR3^+^/T1R3^-^ cells between chow and high-fat diet-fed mice (Fig. S4B).

**Figure 4:**
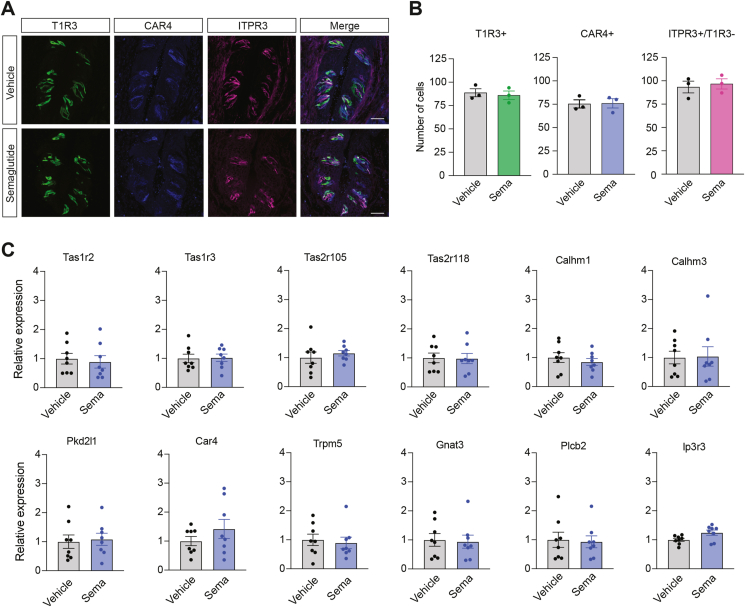
**Chronic semaglutide does not alter the number of cells expressing taste receptors or gene expression of taste receptors and related molecules.** (A) Representative immunohistochemical images of T1R3, CAR4, and ITPR3 in taste buds of the circumvallate papillae (CvP) in vehicle- or semaglutide-treated mice. Scale bar = 40 μm. (B) Quantification of T1R3+ (sweet/umami), CAR4+ (acidic), and ITPR3+/T1R3- (bitter) cells from CvP of vehicle- or semaglutide-treated mice (n = 3/group, unpaired t-tests, all ps = ns). (C) Relative expression of taste-related genes from CvP of vehicle- or semaglutide-treated mice (n = 8/group, unpaired t-tests, all ps = ns). Data are presented as mean ± S.E.M. ns p > 0.05, ∗p < 0.05, ∗∗p < 0.01, ∗∗∗p < 0.001, ∗∗∗∗p < 0.0001. See Supplementary Table 1 for full statistical details.

Next, we investigated whether chronic semaglutide treatment affects the number of taste receptor cells. Across all taste cell populations examined, semaglutide treatment did not alter the number of labeled cells compared to those of vehicle-treated controls (Figure 4A,B). These data indicate that chronic semaglutide does not measurably impact the abundance or representation of major taste receptor cell subtypes within the CvP of high-fat diet-fed mice.

Finally, we examined whether chronic semaglutide treatment alters the expression of genes involved in peripheral taste signaling in the CvP. We quantified expression of key genes for sweet taste detection (*Tas1r2*, *Tas1r3*) [4,33], bitter taste detection (*Tas2r105*, *Tas2r118*) [29], and canonical intracellular taste transduction pathway shared across sweet and bitter taste cells (*Gnat3*, *Plcb2*, *Ip3r3*, *Trpm5*) [4,34]. We also examined genes involved in ATP-mediated neurotransmitter release from taste receptor cells (*Calhm1*, *Calhm3*) [14,35], as well as markers associated with Type III (sour) taste cells (*Car4*, *Pkd2l1*) [4,36]. Again, we first measured gene expression in chow and high-fat diet-fed mice as a reference, and this experiment revealed no differences between groups for all genes examined (Fig. S4C). Similarly, semaglutide treatment in high-fat diet-fed mice did not significantly alter gene expression relative to vehicle-treated controls (Figure 4C). These findings indicate that chronic semaglutide does not influence transcriptional programs governing peripheral taste receptor identity, signal transduction, or neurotransmitter release. Overall, these data support our behavioral studies and further suggest that semaglutide-induced changes in ingestive behavior are unlikely to be mediated by altered taste signaling.

## 4 Discussion

Semaglutide is now used as a first-line pharmacological treatment for obesity due to its robust and sustained effects on body weight [37,38]. Therefore, several studies in humans have attempted to determine whether semaglutide influences taste signaling as part of its mechanism of action, but this work has generated conflicting results. The present study addressed this question by performing systematic taste responsivity tests in mice during chronic semaglutide treatment. Across multiple taste modalities, we found no evidence that semaglutide impairs peripheral taste signaling. In brief-access licking tests, semaglutide-treated mice displayed lick rates similar to vehicle-treated controls for sweet, bitter, sour, salty, and fatty stimuli, indicating that taste-driven orosensory evaluation remains intact under our experimental conditions. Consistent with these behavioral findings, semaglutide did not alter the abundance of taste receptor cell subtypes within the CvP or the expression of genes involved in taste receptor signaling and neurotransmission. Together, these results indicate that chronic semaglutide treatment does not cause detectable alterations in behavioral taste responsivity or detection mechanisms in mice.

Although chronic semaglutide did not affect lick rate – a commonly used index of taste evaluation – it did increase the total number of licks and trials completed for sucrose solutions. These measures incorporate motivational and behavioral engagement components and therefore may reflect changes in these parameters rather than changes in taste sensitivity per se. This paradoxical increase in sugar responsivity is consistent with recent work in rats showing that chronic semaglutide treatment robustly increases intake of low concentrations of sucrose [9]. Several factors may contribute to our observed increase in sucrose responsivity. First, semaglutide-treated mice weighed substantially less than vehicle-treated controls at the time of testing, which could increase motivational drive. This alone does not likely explain our findings, as many reports have indicated reduced desire for food despite weight loss over extended treatment periods [[39], [40], [41], [42], [43]]. However, we also observed non-significant trends towards increased total licking of sour and salty solutions in semaglutide-treated mice that weighed substantially less than controls, and so it should not be ruled out as a contributing factor. Alternately, all of our experiments were conducted in diet-induced obese mice maintained on a high-fat diet. Compared to chow-fed animals, these mice are chronically exposed to a highly palatable food that not only contains fat but also sugar. This palatable maintenance diet – and resultant habituation to an obesogenic environment – may reduce baseline responsiveness to less palatable food [44], such as chow or simple sugar. Under these conditions, semaglutide may modestly enhance the salience or motivational value of sucrose, which is less calorically dense than the high-fat maintenance diet. Indeed, most studies examining GLP-1R agonists and sucrose reward have been conducted in chow-fed animals [21,[45], [46], [47], [48]], in which baseline motivation for sucrose may be higher than in mice maintained on a highly palatable food. We note that the use of diet-induced obese mice may provide a more translationally relevant model, given our modern food environment that is rich in energy-dense, highly palatable foods. Finally, a recent study demonstrated that chronic semaglutide can increase operant responding for certain food rewards in rats, although they consume significantly less of the same food while freely available [49]. The authors of this study speculated that semaglutide may selectively enhance motivation for small, limited-access, palatable food rewards while simultaneously reducing overall food intake. This interpretation also fits with our data set, as sucrose was only available for brief (5-s) periods. It is possible that mice would have consumed less sucrose if it were freely available.

It has been proposed that GLP-1R agonists broadly reduce motivated behavior, because these drugs not only suppress motivation for food intake but also for consumption of alcohol, nicotine, and other drugs of abuse [[50], [51], [52], [53], [54], [55]]. The literature demonstrating reductions in food and drug reward following GLP-1R agonist treatment is highly compelling, and our findings do not contradict this body of work. Rather, our work and others’ [9,49] add to this literature by suggesting that chronic semaglutide does not uniformly suppress motivated behavior across all domains. Specifically, we observed intact responsivity to bitter, sour, salty, and fatty tastants, and modestly increased intake of sucrose. These findings align with real-world data demonstrating that individuals taking GLP-1R agonists continue to eat regularly, albeit less [39,40]. Moreover, individuals treated with GLP-1R agonists generally report improved quality of life, have increased exercise capacity, and engage in occupational and social activities [56,57]. Together, these observations argue against the idea that GLP-1R agonists induce a global suppression of motivation to a level that becomes detrimental to overall wellbeing. This perspective may alleviate concerns that these drugs reduce general motivation to a pathological degree. Notably, concerns about increased suicidality with GLP-1R agonists have been largely mitigated by subsequent analyses [58,59].

Our results that chronic semaglutide does not directly impact taste responsivity are inconsistent with those of certain studies in humans. However, the clinical literature itself is mixed, with studies reporting reductions [10], improvements [11,12], or no changes [13] in taste perception with GLP-1R agonist treatment. Species differences may contribute to discrepancies between the animal and human literature. Further, human studies typically measure subjective taste perception, whereas rodent studies measure behavioral taste responsivity. This poses a limitation to the translational relevance of rodent studies, as they may capture different aspects of taste function. In addition, the existing clinical studies on semaglutide and taste have relatively small sample sizes, lack appropriate control groups, combine multiple GLP-1R agonists within the same analyses, and/or do not report body weight or weight loss across participants – factors that may influence results. In contrast, preclinical studies provide the opportunity to perform highly-controlled, randomized studies of the effects of GLP-1R agonists on taste. Across complementary behavioral, cellular, and molecular analyses, our results indicate that chronic semaglutide does not detectably impair taste function, at least in the assays we used. Instead, these findings support a model in which GLP-1R agonists reshape ingestive behavior through mechanisms independent of primary taste signaling.

A limitation of our findings is that, unlike the bitter, sour, and salty tastants tested, sucrose is caloric and can produce post-ingestive consequences during extended intake. Although brief-access testing minimizes these effects, it cannot eliminate them entirely, and post-ingestive signals could potentially influence licking behavior. Future studies using non-nutritive sweeteners will therefore be important to determine whether semaglutide selectively enhances responding to caloric sugars or more broadly alters intake of sweet substances. Another caveat of our study is that we selected certain timepoints to measure taste responsivity, and this may have implications for our findings. Indeed, future studies may wish to test behavioral responses acutely (i.e., hours, rather than days) after semaglutide injection, as this may yield changes that were not detected in the current study. However, given the long half-life [18] of semaglutide and that it is administered weekly in humans [38], the present findings still provide insight into how chronic semaglutide impacts behavioral taste responsivity. Given current interest in behavioral effects following GLP-1R agonist drug cessation, it would also be interesting to examine taste responsivity after semaglutide washout.

In this study, we assessed taste behavior as well as cellular and molecular markers for key mediators of taste. Our reference data demonstrated that high-fat diet-fed mice have fewer T1R3^+^ taste cells compared to chow-fed mice, although our gene expression data did not substantiate this finding. It is possible that these changes are reflected only at protein expression level, although this is unlikely as high-fat diet has been shown to reduce T1R3 gene expression in rats [31,32]. Nonetheless, the goal of the study was to determine whether chronic semaglutide affects expression of taste signaling molecules within high-fat diet-fed mice, and these data consistently revealed no changes. We note that we did not measure gustatory nerve responses, functional changes in taste receptor function, or central taste processing. Therefore, semaglutide-mediated changes in these processes remain possible.

In conclusion, our findings demonstrate that chronic semaglutide treatment preserves taste responsivity despite robust reductions in food intake and long-term body weight loss. These results suggest that alterations in taste are unlikely to account for the behavioral effects of GLP-1R agonists. Instead, defining how these drugs reshape behavioral engagement and motivational processes will be essential for understanding their broad efficacy across both preclinical models and clinical populations.

## CRediT authorship contribution statement

**Alisha A. Acosta:** Writing – review & editing, Visualization, Methodology, Investigation, Formal analysis, Data curation. **Hillary Ellis:** Writing – review & editing, Methodology, Investigation, Data curation. **Fang-Yu Hsu:** Writing – review & editing, Visualization, Methodology, Investigation, Formal analysis, Data curation. **Karen K. Yee:** Writing – review & editing, Visualization, Methodology, Investigation, Formal analysis, Data curation. **Guillaume de Lartigue:** Writing – review & editing, Supervision, Resources, Methodology, Funding acquisition, Conceptualization. **Amber L. Alhadeff:** Writing – review & editing, Writing – original draft, Visualization, Supervision, Resources, Project administration, Methodology, Funding acquisition, Formal analysis, Conceptualization.

## Declaration of competing interest

A.L.A. is a scientific advisory board member for Zealand Pharma. This studies is unrelated to the work presented in the current manuscript.

## Data Availability

Data will be made available on request.

## Appendix A Supplementary data

Supplementary Table 1 Statistics for all main and supplementary figures.

## References

[1] Drucker D.J. (2022). GLP-1 physiology informs the pharmacotherapy of obesity. Mol Metabol.

[2] Williams D.L. (2022). The diverse effects of brain glucagon-like peptide 1 receptors on ingestive behaviour. Br J Pharmacol.

[3] Kanoski S.E., Hayes M.R., Skibicka K.P. (2016). GLP-1 and weight loss: unraveling the diverse neural circuitry. Am J Physiol Regul Integr Comp Physiol.

[4] Chandrashekar J., Hoon M.A., Ryba N.J., Zuker C.S. (2006). The receptors and cells for Mammalian taste. Nature.

[5] Shin Y.K., Martin B., Golden E., Dotson C.D., Maudsley S., Kim W. (2008). Modulation of taste sensitivity by GLP-1 signaling. J Neurochem.

[6] Martin B., Dotson C.D., Shin Y.K., Ji S., Drucker D.J., Maudsley S. (2009). Modulation of taste sensitivity by GLP-1 signaling in taste buds. Ann N Y Acad Sci.

[7] Takai S., Yasumatsu K., Inoue M., Iwata S., Yoshida R., Shigemura N. (2015). Glucagon-like peptide-1 is specifically involved in sweet taste transmission. FASEB J.

[8] Treesukosol Y., Moran T.H. (2022). Administration of Exendin-4 but not CCK alters lick responses and trial initiation to sucrose and intralipid during brief-access tests. Chem Senses.

[9] Cawthon C.R., Blonde G.D., Nisi A.V., Bloomston H.M., Krubitski B., le Roux (2023). Chronic semaglutide treatment in rats leads to daily excessive concentration-dependent sucrose intake. J Endocr Soc.

[10] Khan R., Doty R.L. (2025). GLP-1 receptor agonists significantly impair taste function. Physiol Behav.

[11] Jensterle M., Kovac J., Vovk A., Ferjan S., Battelino S., Battelino T. (2025). Semaglutide and taste in women with obesity and polycystic ovary syndrome: a randomized placebo-controlled study. J Clin Endocrinol Metab.

[12] Brindsi M.C., Brondel L., Meillon S., Barthet S., Grall S., Fenech C. (2019). Proof of concept: effect of GLP-1 agonist on food hedonic responses and taste sensitivity in poor controlled type 2 diabetic patients. Diabetes Metabol Syndr.

[13] Kapan A., Moser O., Felsinger R., Waldhoer T., Haider S. (2025). Real-world insights into incretin-based therapy: associations between changes in taste perception and appetite regulation in individuals with obesity and overweight: a cross-sectional study. Diabetes Obes Metabol.

[14] Taruno A., Vingtdeux V., Ohmoto M., Ma Z., Dvoryanchikov G., Li A. (2013). CALHM1 ion channel mediates purinergic neurotransmission of sweet, bitter and umami tastes. Nature.

[15] Glendinning J.I., Bloom L.D., Onishi M., Zheng K.H., Damak S., Margolskee R.F. (2005). Contribution of alpha-gustducin to taste-guided licking responses of mice. Chem Senses.

[16] Glendinning J.I., Gresack J., Spector A.C. (2002). A high-throughput screening procedure for identifying mice with aberrant taste and oromotor function. Chem Senses.

[17] Sinclair M.S., Perea-Martinez I., Abouyared M., St John S.J., Chaudhari N. (2015). Oxytocin decreases sweet taste sensitivity in mice. Physiol Behav.

[18] Yang X.D., Yang Y.Y. (2024). Clinical pharmacokinetics of semaglutide: a systematic review. Drug Des Dev Ther.

[19] Gabery S., Salinas C.G., Paulsen S.J., Ahnfelt-Ronne J., Alanentalo T., Baquero A.F. (2020). Semaglutide lowers body weight in rodents via distributed neural pathways. JCI Insight.

[20] Shah H., Ayala J.E. (2026). Prolonged semaglutide treatment reveals stage-dependent changes to feeding behavior and metabolic adaptations in Male mice. Diabetes.

[21] Ghidewon M., Wald H.S., McKnight A.D., De Jonghe, Breen D.M., Alhadeff A.L. (2022). Growth differentiation factor 15 (GDF15) and semaglutide inhibit food intake and body weight through largely distinct, additive mechanisms. Diabetes Obes Metabol.

[22] Huang K.P., Acosta A.A., Ghidewon M.Y., McKnight A.D., Almeida M.S., Nyema N.T. (2024). Dissociable hindbrain GLP1R circuits for satiety and aversion. Nature.

[23] Blonde G.D., Spector A.C. (2020). Masking the detection of taste stimuli in rats: NaCl and sucrose. Chem Senses.

[24] Roper S.D., Chaudhari N. (2017). Taste buds: cells, signals and synapses. Nat Rev Neurosci.

[25] Mattes R.D. (2009). Is there a fatty acid taste?. Annu Rev Nutr.

[26] Besnard P., Passilly-Degrace P., Khan N.A. (2016). Taste of fat: a sixth taste modality?. Physiol Rev.

[27] Bachmanov A.A., Beauchamp G.K. (2007). Taste receptor genes. Annu Rev Nutr.

[28] Chandrashekar J., Yarmolinsky D., von Buchholtz L., Oka Y., Sly W., Ryba N. (2009). The taste of carbonation. Science.

[29] Adler E., Hoon M.A., Mueller K.L., Chandrashekar J., Ryba N.J., Zuker C.S. (2000). A novel family of Mammalian taste receptors. Cell.

[30] Ohmoto M., Jyotaki M., Foskett J.K., Matsumoto I. (2020). Sodium-taste cells require Skn-1a for generation and share molecular features with sweet, umami, and bitter taste cells. eNeuro.

[31] Chen K., Yan J., Suo Y., Li J., Wang Q., Lv B. (2010). Nutritional status alters saccharin intake and sweet receptor mRNA expression in rat taste buds. Brain Res.

[32] Zhang X.J., Wang Y.Q., Long Y., Wang L., Li Y., Gao F. (2013). Alteration of sweet taste in high-fat diet induced Obese rats after 4 weeks treatment with exenatide. Peptides.

[33] Nelson G., Hoon M.A., Chandrashekar J., Zhang Y., Ryba NJ., Zuker C.S. (2001). Mammalian sweet taste receptors. Cell.

[34] Zhang Y., Hoon M.A., Chandrashekar J., Mueller K.L., Cook B., Wu D. (2003). Coding of sweet, bitter, and umami tastes: different receptor cells sharing similar signaling pathways. Cell.

[35] Ma Z., Taruno A., Ohmoto M., Jyotaki M., Lim J.C., Miyazaki H. (2018). CALHM3 is essential for rapid ion channel-mediated purinergic neurotransmission of GPCR-mediated tastes. Neuron.

[36] Huang A.L., Chen X., Hoon M.A., Chandrashekar J., Guo W., Trankner D. (2006). The cells and logic for mammalian sour taste detection. Nature.

[37] Wadden T.A., Bailey T.S., Billings L.K., Davies M., Frias J.P., Koroleva A. (2021). Effect of subcutaneous semaglutide vs placebo as an adjunct to intensive behavioral therapy on body weight in adults with overweight or obesity: the STEP 3 randomized clinical trial. JAMA.

[38] Wilding J.P.H., Batterham R.L., Calanna S., Davies M., Van Gaal L.F., Lingvay I. (2021). Once-weekly semaglutide in adults with overweight or obesity. N Engl J Med.

[39] Blundell J., Finlayson G., Axelsen M., Flint A., Gibbons C., Kvist T. (2017). Effects of once-weekly semaglutide on appetite, energy intake, control of eating, food preference and body weight in subjects with obesity. Diabetes Obes Metabol.

[40] Gibbons C., Blundell J., Hoff S.T., Dahl K., Bauer R., Baekdal T. (2021). Effects of oral semaglutide on energy intake, food preference, appetite, control of eating and body weight in subjects with type 2 diabetes. Diabetes Obes Metabol.

[41] Kadouh H., Chedid V., Halawi H., Burton D.D., Clark M.M., Khemani D. (2020). GLP-1 analog modulates appetite, taste preference, gut hormones, and regional body fat stores in adults with obesity. J Clin Endocrinol Metab.

[42] Wharton S., Batterham R.L., Bhatta M., Buscemi S., Christensen L.N., Frias J.P. (2023). Two-year effect of semaglutide 2.4 mg on control of eating in adults with overweight/obesity: step 5. Obesity.

[43] Friedrichsen M., Breitschaft A., Tadayon S., Wizert A., Skovgaard D. (2021). The effect of semaglutide 2.4 mg once weekly on energy intake, appetite, control of eating, and gastric emptying in adults with obesity. Diabetes Obes Metabol.

[44] Mazzone C.M., Liang-Guallpa J., Wolcott N.S., Boone M.H., Southern M., Kobzar N.P. (2020). High-fat food biases hypothalamic and mesolimbic expression of consummatory drives. Nat Neurosci.

[45] Dickson SL., Shirazi R.H., Hansson C., Bergquist F., Nissbrandt H., Skibicka K.P. (2012). The glucagon-like peptide 1 (GLP-1) analogue, exendin-4, decreases the rewarding value of food: a new role for mesolimbic GLP-1 receptors. J Neurosci.

[46] Ong Z.Y., Liu J.J., Pang Z.P., Grill H.J. (2017). Paraventricular thalamic control of food intake and reward: role of glucagon-like Peptide-1 receptor signaling. Neuropsychopharmacology.

[47] Alhadeff A.L., Grill H.J. (2014). Hindbrain nucleus tractus solitarius glucagon-like peptide-1 receptor signaling reduces appetitive and motivational aspects of feeding. Am J Physiol Regul Integr Comp Physiol.

[48] Colvin K.J., Killen H.S., Kanter M.J., Halperin M.C., Engel L., Currie P.J. (2020). Brain site-specific inhibitory effects of the GLP-1 analogue Exendin-4 on alcohol intake and operant responding for palatable food. Int J Mol Sci.

[49] Chang S.E., Turner C.A., Pagan N.M., Pereira D., Kleer S., Flagel S.B. (2026). Chronic semaglutide treatment enhances the incentive motivational value of a small food reward and associated cue in male and female rats. Psychopharmacology (Berl).

[50] Schmidt H.D., Mietlicki-Baase E.G., Ige K.Y., Maurer J.J., Reiner D. ., Zimmer D.J. (2016). Glucagon-like Peptide-1 receptor activation in the ventral tegmental area decreases the reinforcing efficacy of cocaine. Neuropsychopharmacology.

[51] Tuesta L.M., Chen Z., Duncan A., Fowler C.D., Ishikawa C.D., Lee B.R. (2017). GLP-1 acts on habenular avoidance circuits to control nicotine intake. Nat Neurosci.

[52] Douton J.E., Horvath N., Mills-Huffnagle S., Nyland J.E., Hajnal A., Grigson P.S. (2022). Glucagon-like peptide-1 receptor agonist, liraglutide, reduces heroin self-administration and drug-induced reinstatement of heroin-seeking behaviour in rats. Addict Biol.

[53] Falk S., Petersen J., Svendsen C., Romero-Leguizamon C.R., Jorgensen S.H., Krauth N. (2023). GLP-1 and nicotine combination therapy engages hypothalamic and mesolimbic pathways to reverse obesity. Cell Rep.

[54] Skibicka K.P. (2013). The central GLP-1: implications for food and drug reward. Front Neurosci.

[55] Farokhnia M., Tazare J., Pince C.L., Bruns N., Gray J.C., Lo Re V. (2025). Glucagon-like peptide-1 receptor agonists, but not dipeptidyl peptidase-4 inhibitors, reduce alcohol intake. J Clin Investig.

[56] Kosiborod M.N., Verma S., Borlaug B.A., Butler J., Davies M.J., Jon Jonsen T. (2024). Effects of semaglutide on symptoms, function, and quality of life in patients with heart failure with preserved ejection fraction and obesity: a prespecified analysis of the STEP-HFpEF trial. Circulation.

[57] Billings L.K., Handelsman Y., Heile M., Schneider D., Wyne K. (2018). Health-related quality of life assessments with once-weekly glucagon-like Peptide-1 receptor agonists in type 2 diabetes mellitus. J Manag Care Spec Pharm.

[58] Shapiro S.B., Yin H., Yu O.H.Y., Rej S., Suissa S., Azoulay L. (2025). Glucagon-like peptide-1 receptor agonists and risk of suicidality among patients with type 2 diabetes: active comparator, new user cohort study. BMJ.

[59] Wang W., Volkow N.D., Berger N.A., Davis P.B., Kaelber D.C., Xu R. (2024). Association of semaglutide with risk of suicidal ideation in a real-world cohort. Nat Med.

