# Supplementary material for: Chronic semaglutide alters ingestive behavior without impairing taste function in mice
Source: Mol Metab. 2026 Jun 27;110:102410. doi: 10.1016/j.molmet.2026.102410 (PMC13382136; doi:10.1016/j.molmet.2026.102410)
Supplement: Multimedia component 2 [file mmc2.docx]

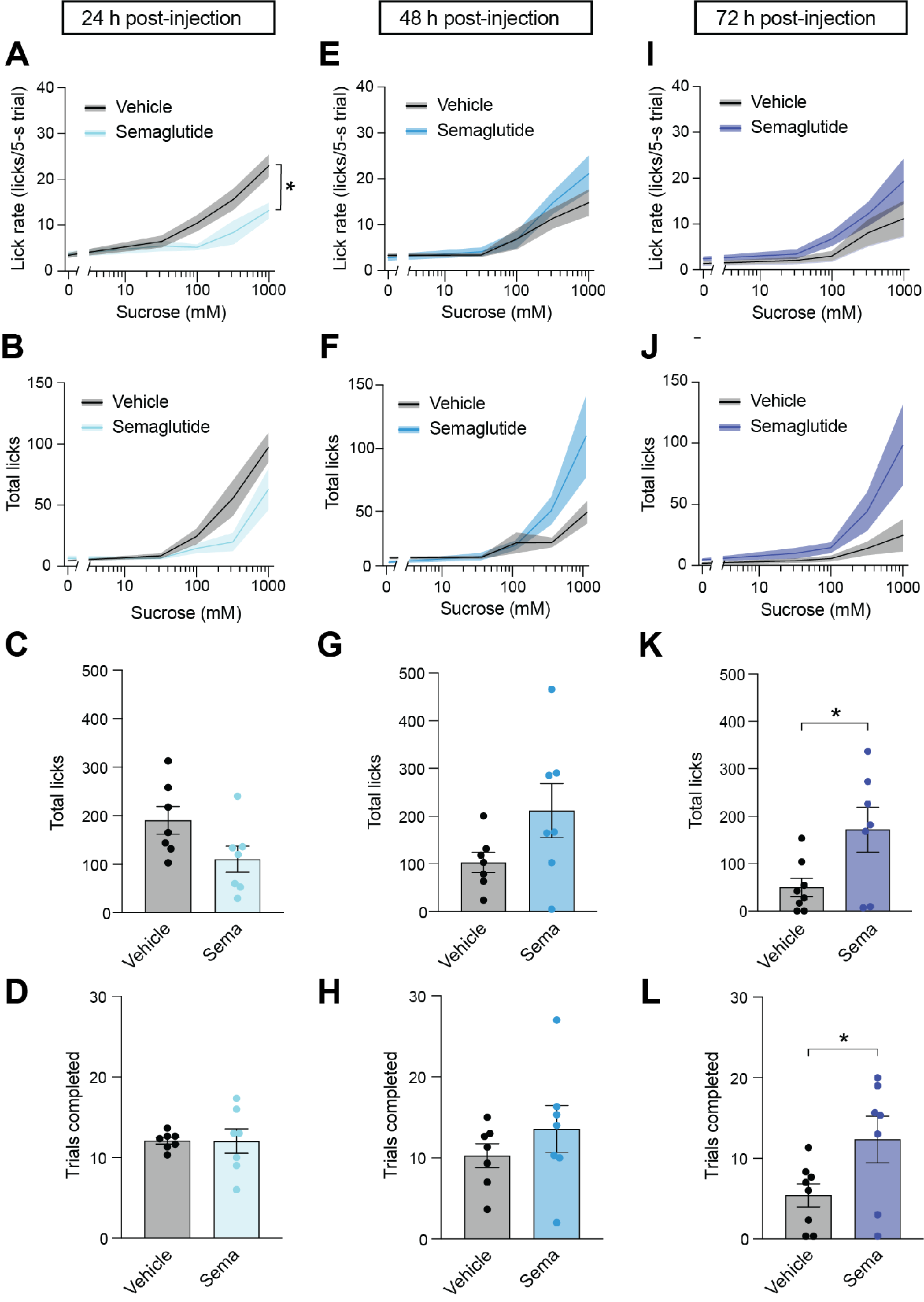


**Figure S1. Effects of semaglutide on sweet taste responsivity at 24 h, 48 h, and 72 h post-injection**

(A, E, I) Lick rate across sucrose concentrations at 24 h (A, n=7/group, two-way repeated measures ANOVA, p<0.05), 48 h (E, n=7/group, two-way repeated measures ANOVA, p=ns), and 72 h (I, n=7-8/group, two-way repeated measures ANOVA, p=ns) post vehicle and semaglutide injections.

(B, F, J) Total licks across sucrose concentrations at 24 h (B, n=7/group, two-way repeated measures ANOVA, p=ns), 48 h (F, n=7/group, two-way repeated measures ANOVA, p=ns), and 72 h (J, n=7-8/group, two-way repeated measures ANOVA, p=ns) post vehicle and semaglutide injections.

(C, G, K) Total licks per session at 24 h (C, n=8/group, unpaired t-test, p=ns), 48 h (G, n=7-8/group, unpaired t-test, p=ns), and 72 h (K, n=8/group, unpaired t-test, p<0.05) post vehicle and semaglutide injections.

(D, H, L) Total trials completed at 24 h (D, n=8/group, unpaired t-test, p=ns), 48 h (H, n=7-8/group, unpaired t-test, p=ns), and 72 h (L, n=8/group, unpaired t-test, p<0.05) post vehicle and semaglutide injections.

Data are presented as mean ± S.E.M. ns p>0.05, *p<0.05, **p<0.01, ***p<0.001, ****p<0.0001. See Supplementary Table 1 for full statistical details.


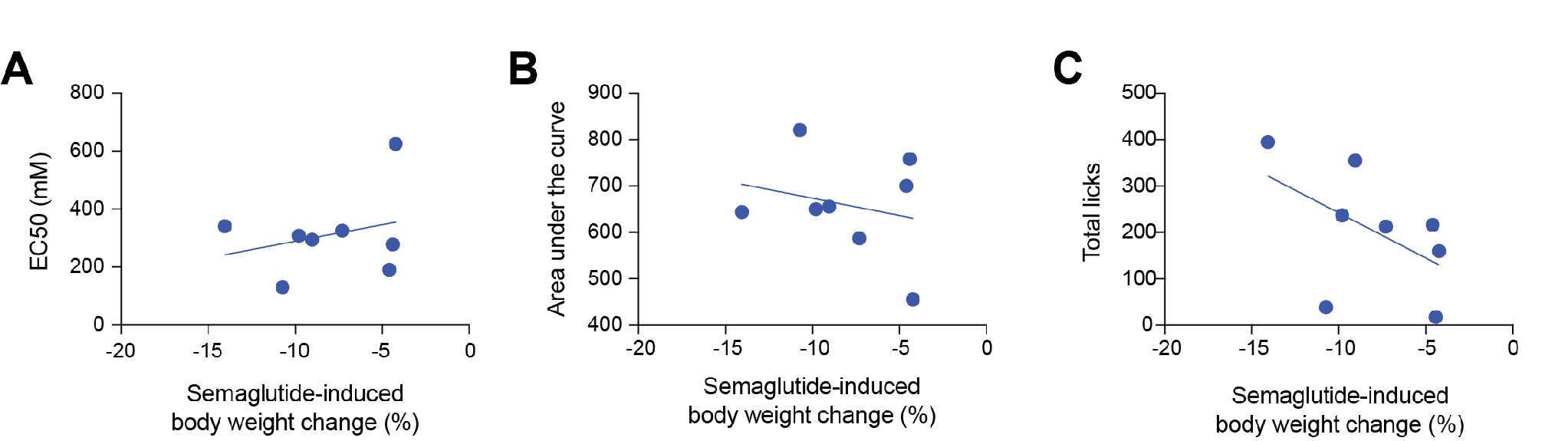


**Figure S2. Correlations between taste sensitivity parameters and semaglutide-induced body weight change**

(A) Correlation between EC50 and semaglutide-induced body weight change (related to data in Figure 2C) (n=8, Pearson correlation, r^2^=0.078, p=ns).

(B) Correlation between area under the curve and semaglutide-induced body weight change (related to data in Figure 2D) (n=8, Pearson correlation, r^2^=0.057, p=ns).

(C) Correlation between total licks and semaglutide-induced body weight change (related to data in Figure 2E) (n=8, Pearson correlation, r^2^=0.27, p=ns).

ns p>0.05, *p<0.05, **p<0.01, ***p<0.001, ****p<0.0001. See Supplementary Table 1 for full statistical details.


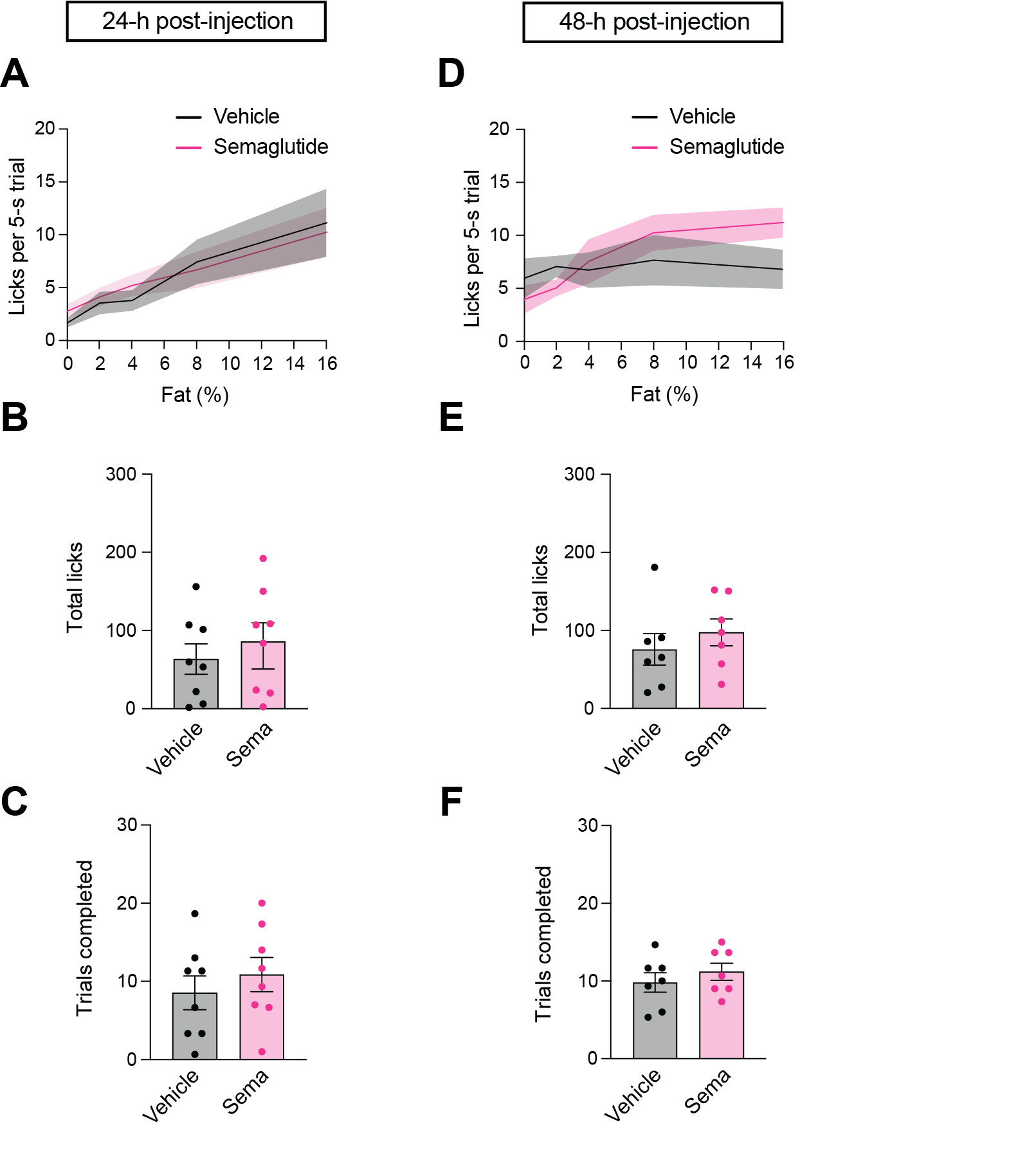


**Figure S3. Effects of semaglutide on fat taste responsivity at 24 h and 48 h post-injection**

(A, D) Lick rate across fat concentrations at 24 h (A, n=8/group, two-way repeated measures ANOVA, p=ns) and 48 h (D, n=7/group, two-way repeated measures ANOVA, p=ns) post vehicle and semaglutide injections.

(B, E) Total licks per session at 24 h (B, n=8/group, unpaired t-test, p=ns) and 48 h (E, n=7/group, unpaired t-test, p=ns) post vehicle and semaglutide injections.

(C, F) Total trials completed at 24 h (C, n=8/group, unpaired t-test, p=ns) and 48 h (F, n=7/group, unpaired t-test, p=ns) post vehicle and semaglutide injections.

Data are presented as mean ± S.E.M. ns p>0.05, *p<0.05, **p<0.01, ***p<0.001, ****p<0.0001. See Supplementary Table 1 for full statistical details.

**
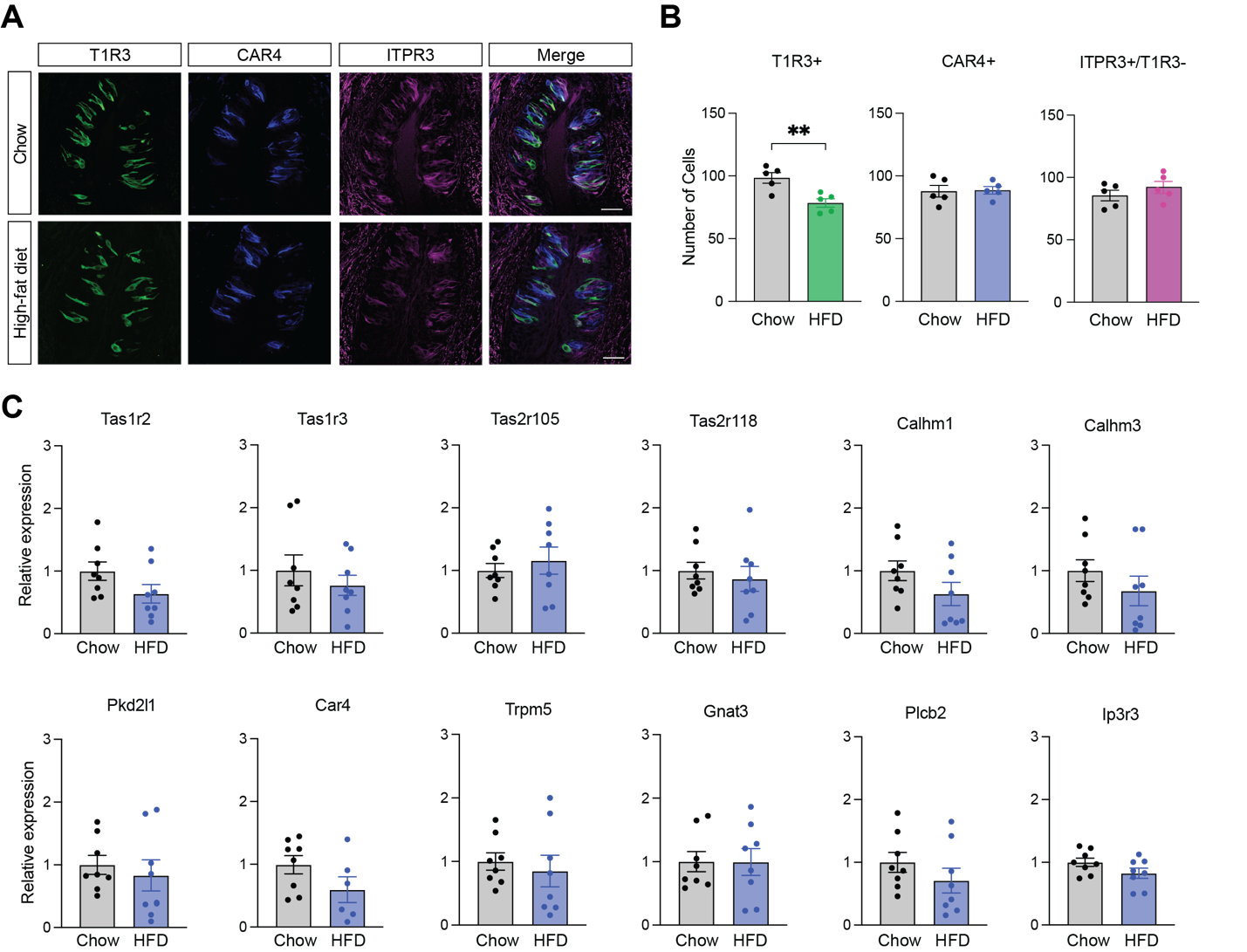
**

**Figure S4. Number of cells expressing taste receptors and gene expression of taste receptors and related molecules in chow-fed and high-fat diet (HFD)-fed mice**

(A) Representative immunohistochemical images of T1R3, CAR4, and ITPR3 in taste buds of the circumvallate papillae (CvP) in chow-fed or high-fat diet-fed mice. Scale bar = 40 μm.

(B) Quantification of T1R3+ (sweet/umami), CAR4+ (acidic), and ITPR3+/T1R3- (bitter) cells from CvP of chow-fed or high-fat diet-fed mice (n=5/group, unpaired t-tests, p<0.01 for T1R3+ cells, p=ns for others).

(C) Relative gene expression of taste-related genes from CvP of chow-fed or high-fat diet-fed mice (n=6-8/group, all ps=ns).

Data are presented as mean ± S.E.M. ns p>0.05, *p<0.05, **p<0.01, ***p<0.001, ****p<0.0001. See Supplementary Table 1 for full statistical details.
